# Structure, function, and regulation of mitofusin‐2 in health and disease

**DOI:** 10.1111/brv.12378

**Published:** 2017-10-25

**Authors:** Gursimran Chandhok, Michael Lazarou, Brent Neumann

**Affiliations:** ^1^ Department of Anatomy and Developmental Biology, and Neuroscience Program, Monash Biomedicine Discovery Institute Monash University Melbourne Victoria 3800 Australia; ^2^ Department of Biochemistry and Molecular Biology, and Neuroscience Program, Monash Biomedicine Discovery Institute Monash University Melbourne Victoria 3800 Australia

**Keywords:** mitofusin‐2, mitofusin‐1, mitochondria, mitochondrial dynamics, Charcot–Marie–Tooth disease, neurodegenerative disease, diabetes, obesity, vascular disease

## Abstract

Mitochondria are highly dynamic organelles that constantly migrate, fuse, and divide to regulate their shape, size, number, and bioenergetic function. Mitofusins (Mfn1/2), optic atrophy 1 (OPA1), and dynamin‐related protein 1 (Drp1), are key regulators of mitochondrial fusion and fission. Mutations in these molecules are associated with severe neurodegenerative and non‐neurological diseases pointing to the importance of functional mitochondrial dynamics in normal cell physiology. In recent years, significant progress has been made in our understanding of mitochondrial dynamics, which has raised interest in defining the physiological roles of key regulators of fusion and fission and led to the identification of additional functions of Mfn2 in mitochondrial metabolism, cell signalling, and apoptosis. In this review, we summarize the current knowledge of the structural and functional properties of Mfn2 as well as its regulation in different tissues, and also discuss the consequences of aberrant Mfn2 expression.

## I. INTRODUCTION: THE ESSENTIAL ROLE OF MITOFUSIN‐2 IN HUMAN PHYSIOLOGY

Mitochondria are commonly referred to as the powerhouses of the cell, producing most of the ATP required for normal metabolic processes. Mitochondria are involved in essential physiological processes including energy production *via* the Krebs cycle, fatty acid metabolism and gluconeogenesis, calcium buffering, and apoptosis.

The essential functions of mitochondria have been attributed to their unique dynamic nature: the ability to undergo continuous cycles of fusion and fission that result in changes in mitochondrial morphology and movement of these organelles along the cytoskeleton. These events ultimately lead to branched or tubular mitochondrial networks that allow active communication and sharing of contents between mitochondrial compartments, as well as with other organelles such as the endoplasmic reticulum, to meet the energy demands of cells, regulate Ca^2+^ influx and, cope with oxidative damage (Westermann, 2012). Mitochondrial dynamics allow for the even distribution of unique mitochondrial components like the inner membrane lipid cardiolipin, for genetic complementation, for the transfer of mitochondria to daughter cells during cell division, and for the regulation of apoptosis through segregation of damaged mitochondria (Mishra & Chan, 2014; Schlattner *et al.,* 2014).

Mitochondrial fusion and fission typically counterbalance each other, but environmental stimuli, developmental status, and metabolic demands of the cells can shift the balance towards either fission or fusion. Although mitochondria are essential for all cell types, they are particularly important for highly specialized cell types, such as neurons and sperm, which require increased levels of energy and Ca^2+^ regulation (Rizzuto *et al.,* 2012). Importantly, neurons also require mitochondria to be trafficked for long distances along neurites, placing increased demand on their migration capacity and shape. Recent research has begun shedding light on the molecules and mechanisms that control these processes, with mitofusins (Mfn1 and Mfn2), Optic atrophy 1 (OPA1), and dynamin‐related protein 1 (Drp1) found to be key mediators of mitochondrial fusion and fission.

As mitochondria play crucial roles in meeting a cell's energy demands, it is inevitable that defects in mitochondria can lead to deleterious effects on cellular health, ultimately causing cell injury and death. There is growing evidence that defective mitochondrial function is associated with severe neurodegenerative conditions such as Parkinson's, Huntington's, Alzheimer's, and Charcot–Marie–Tooth diseases (Lu, 2009). Mitofusin dysregulation is also linked to metabolic disorders, including obesity and type‐2 diabetes (Cheng & Almeida, 2014). However, the exact mechanisms behind how mitochondrial pathways affect physiological function are yet to be determined. In this review, we focus on the physiological role of Mfn2, the molecular mechanisms behind its function and regulation, and its role in human disease.

## II. MITOCHONDRIAL DYNAMICS

### (1) Mitochondrial fusion

Mitochondrial fusion is a process whereby the outer and inner mitochondrial membranes of two originally distinct mitochondria physically merge into one. The fusion process enables the exchange of contents between mitochondria, allowing defective organelles to regain essential components of the respiratory chain and mitochondrial DNA (Detmer & Chan, 2007b). The first experimental evidence of mitochondrial fusion was in *Saccharomyces cerevisiae* (Nunnari *et al.,* 1997), where two different haploid yeast cell populations containing mitochondria labelled with either green or red fluorescence were mated, and the green and red mitochondria redistributed and merged in the diploid zygote. The first known mediator of mitochondrial fusion, the *fuzzy onion* (*fzo*) gene (which encodes the founding member of the conserved mitofusin GTPase family, for which the mammalian homologs are Mfn1 and Mfn2) was isolated from a genetic screening approach in the fruit fly *Drosophila melanogaster* (Hales & Fuller, 1997).

**Figure 1:**
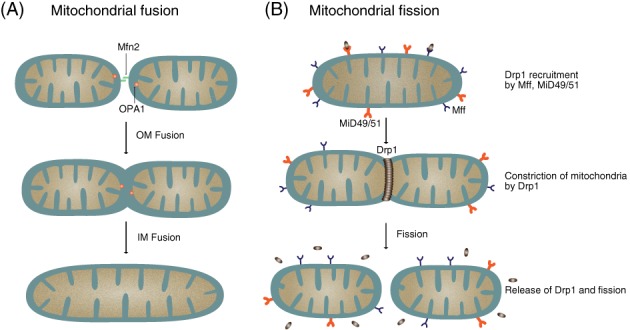
Mitochondrial dynamics. (A) Mitochondrial fusion is a two‐step process that requires fusion of both outer (OM) and inner (IM) mitochondrial membranes mediated by mitofusins Mfn1/2 and optic atrophy 1 (OPA1), respectively. (B) Mitochondrial fission involves recruitment of dynamin‐related protein 1 (Drp1) by mitochondrial fission factor (Mff) and mitochondrial dynamics proteins (MiDs), followed by constriction of Drp1 on the outer mitochondrial membrane. Recent evidence has demonstrated that following partial constriction by Drp1, dynamin 2 mediates the final constriction of the mitochondrial membrane to achieve fission (Lee et al., 2016) (not shown in figure).

Mitofusins (Mfn1/2) on the outer mitochondrial membrane and OPA1 on the inner mitochondrial membrane are central to the fusion process (Fig. 1A). Since mitochondria are double‐membrane organelles, full fusion requires merging of both the outer and inner membranes. Outer membrane fusion is mediated by the mitofusins, large GTPases that traverse the outer mitochondrial membrane twice, with the amino and carboxy termini both facing into the cytoplasm (Alexander *et al.,* 2000). Mitofusins form both homo‐oligomeric (Mfn1–Mfn1 or Mfn2–Mfn2) and hetero‐oligomeric (Mfn1–Mfn2) complexes in trans between apposing mitochondria (Chen *et al.,* 2003; Griffin & Chan, 2006; Qi *et al.,* 2016). Prior to fusion, curving of the outer membranes is promoted by the hydrolysis of cardiolipin to phosphatidic acid, a process mediated by phospholipase‐D (Choi *et al.,* 2006). Once mitofusins are tethered, hydrolysis of GTP enables mitochondrial fusion (Ryan & Stojanovski, 2012).

The importance of the mitofusins is highlighted by the phenotypes observed in knockout mouse strains lacking either one or both of the proteins (Chan, 2006). Although loss of either Mfn1 or Mfn2 causes lethality in mice, cultured cells from these animals can be obtained and they display overtly fragmented mitochondria (Chen *et al.,* 2003). Interestingly, and as discussed in greater detail below, mutations in human Mfn2 but not Mfn1 leads to Charcot–Marie–Tooth disease type 2A, a neurodegenerative disorder characterized by progressive sensory and motor losses in the limbs (Zuchner *et al.,* 2004; Misko *et al.,* 2012; Rouzier *et al.,* 2012; Bouhy & Timmerman, 2013).

Fusion of the inner mitochondrial membrane requires optic atrophy 1 (OPA1), a large GTPase tethered to the inner mitochondrial membrane facing the intermembrane space (Olichon *et al.,* 2002; Cipolat *et al.,* 2004). Localization of the yeast OPA1 ortholog (Mgm1) to the intermembrane space is only required for inner mitochondrial membrane fusion (Meeusen, McCaffery & Nunnari, 2004). The function of OPA1 in fusion requires Mfn1, but interestingly not Mfn2 (Cipolat *et al.,* 2004). Importantly, fusion of the inner mitochondrial membrane appears to rely on the mitochondrial membrane potential, whereas fusion of the outer mitochondrial membrane requires high levels of GTP (Meeusen *et al.,* 2004). In addition to inner membrane fusion, OPA1 is involved in maintaining the shape of mitochondrial cristae (Olichon *et al.,* 2003; Frezza *et al.,* 2006; Cogliati *et al.,* 2013; Patten *et al.,* 2014). As a result, OPA1 has a direct metabolic effect by stabilizing respiratory chain supercomplexes (Cogliati *et al.,* 2013). Defects in either or both processes underlie the progressive blindness disorder dominant optic atrophy, which is caused by *OPA1* mutations in humans (Alexander *et al.,* 2000; Delettre *et al.,* 2000).

### (2) Mitochondrial fission

Mitochondrial fission is a process by which a single mitochondrion is divided into two. The fission process is necessary for remodelling and rearrangement of mitochondrial networks, proper mitochondrial transport, facilitating apoptosis, and for the removal of dysfunctional mitochondria (Westermann, 2012). Mitochondrial fission requires recruitment of Drp1, a large dynamin‐related GTPase from the cytosol (Fig. 1B). Drp1 proteins physically associate with one another, forming curved structures that wrap around the entire outer mitochondrial surface, constricting them, and then using the energy from GTP hydrolysis to pinch off the mitochondria (Bleazard *et al.,* 1999; Legesse‐Miller, Massol & Kirchhausen, 2003; Friedman & Nunnari, 2014). Recent evidence in mammalian cells has defined the critical role of the dynamin 2 protein in the fission process (Lee *et al.,* 2016). Lee *et al*. (2016) demonstrated that fission occurs through a two‐step process, with Drp1 initially providing partial constriction of the mitochondrial membrane, and dynamin 2 subsequently binding and constricting further to split the organelle into two.

Since Drp1 lacks a membrane‐binding domain, Drp1 recruitment to the fission site involves multiple molecules, including the mitochondrial fission factor (Mff), and the mitochondrial dynamics proteins of 49 and 51 kDa (MiD49 and MiD51) (Fig. 1B) (Otera *et al.,* 2010; Osellame *et al.,* 2016). Overexpression of Mff induces the recruitment of Drp1 to mitochondria and subsequent mitochondrial fragmentation, whereas its deficiency leads to mitochondrial elongation (Otera *et al.,* 2010). MiD49 and MiD51 are outer mitochondrial membrane tethered proteins that share 45% sequence identity and directly recruit Drp1 to mitochondria (Palmer *et al.,* 2011; Zhao *et al.,* 2011). Reduced or enhanced expression of these proteins promotes fusion, due to decreased Drp1 association with mitochondria, or the sequestration of Drp1 at the mitochondria surface, respectively (Palmer *et al.,* 2011, 2013). In addition, gain‐of‐function of ganglioside‐induced differentiation‐associated protein 1 (GDAP1) has been shown to induce mitochondrial fragmentation, while its loss‐of‐function promotes mitochondrial elongation (Niemann *et al.,* 2005). Finally, fission protein 1 homolog (Fis1) was also previously implicated in Drp1 recruitment, although mammalian cells lacking this protein have minimal or no fission defects (Otera *et al.,* 2010; Loson *et al.,* 2013). More recent evidence supports an alternative role for Fis1 in mitophagy (Shen *et al.,* 2014; Yamano *et al.,* 2014).

## III. GENOMIC AND PROTEIN ORGANIZATION OF THE MITOFUSINS

**Figure 2:**
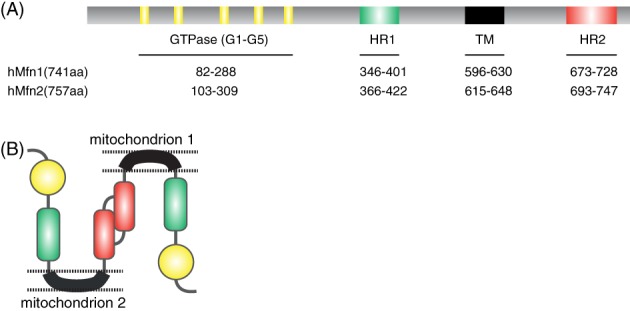
The functional domains of mitofusin‐2 (Mfn2). (A) The GTPase domains consists of five G motifs (G1–G5) shown in yellow. Heptad repeat (HR) coiled‐coil regions 1 and 2 are shown in green and red, respectively. The transmembrane (TM) domain is shown in black. (B) Mfn2 interacts in trans forming either homotypic or heterotypic (with Mfn1) dimers to produce mitochondrial tethering that precedes mitochondrial fusion.

Mammalian mitochondrial fusion proteins were identified as human homologs of *Drosophila* fuzzy onions protein (Fzo) (Hales & Fuller, 1997). Mfn1 [741 amino acids (aa)] and Mfn2 (757 aa) are transmembrane GTPases located on the outer mitochondrial membrane that share 63% homology with the same relevant functional domains. The conserved domains consist of an amino‐terminal GTP binding domain and coiled‐coil domain (heptad repeat HR1), and a carboxy‐terminal with a bipartite transmembrane domain and a second coiled‐coil domain (heptad repeat HR2) (Fig. 2A). Both the GTPase and coiled‐coil domains are exposed to the cytosol. In addition, Mfn2 possesses an N‐terminal Ras‐binding domain that is absent in Mfn1, suggesting specific roles of Mfn2 (Chen *et al.,* 2004).

The GTPase domain has five functional motifs, G1–G5, that each has a specific function. G1 binds the phosphate of the GTP molecule; G3 coordinates the Mg^2+^ needed for hydrolysis; G1, G2 and G3 together form the catalytic centre; G4 and G5 provide the specific conformation required for GTP binding (Bourne, Sanders & McCormick, 1990, 1991). The HR2 domain is involved in the tethering of two adjacent mitochondria through a dimeric antiparallel coiled‐coil structure forming either homotypic (Mfn1–Mfn1 or Mfn2–Mfn2) or heterotypic (Mfn1–Mfn2) dimers (Fig. 2B) (Chen et al., 2003; Koshiba et al., 2004). Recently, two independent studies have shed novel insights into Mfn structure and mitochondrial tethering (Qi et al., 2016; Cao et al., 2017). Crystal structures were generated from Mfn1 constructs containing an internal deletion of the second helical bundle and predicted transmembrane (TM) regions (Qi et al., 2016; Cao et al., 2017). The proposed model for fusion involves rotation of HB1 (helix bundle 1 extending from the GTPase domain) upon GTP hydrolysis, which allows HB2 (helix bindle 2 extending from the C terminal) to bend and attach to the GTPase domain, thereby bringing opposing mitochondrial membranes together (Qi et al., 2016). Both the GTPase and HR2 domains are crucial for the fusion process as mutations in these domains either prevent GTPase activity and thereby eliminate fusogenic activity, or disrupt the dimeric antiparallel coiled‐coil structure and abolish membrane tethering (Qi et al., 2016). Consistent with this, Cao et al. (2017) observed that dimerization of the GTPase domains is mediated by a conserved GTPase domain interface across the nucleotide binding site, resulting in activation of GTPase activity. Preventing dimerization of the GTPase domain led to impaired fusogenic activity of Mfn1, suggesting a crucial role for the G‐interface in mitochondrial fusion (Cao et al., 2017).

Mfn2 activity is regulated by the transcriptional modulator Smad2, and the guanine nucleotide exchange factor (GEF) Rab and Ras Interactor 1 (RIN1) (Kumar et al., 2016). Smad2 was shown to act as a scaffold to recruit RIN1 into a complex with Mfn2. The Smad2–RIN1–Mfn2 complex allows RIN1 to act as a GEF for Mfn2‐GTPase activation, which promotes mitochondrial fusion (Kumar et al., 2016). Recent evidence has provided new views on Mfn2 organization and plasticity. Mfn2 was shown to exist in two functionally distinct conformations, a compressed (inactive) and extended (active) form, directed by specific intramolecular interactions (Franco et al., 2016). This has changed the previous view that Mfn2 exists only in an active state and thereby provides strong evidence that Mfn2 activity is tightly regulated.

## IV. REGULATION OF MITOFUSIN EXPRESSION

Expression levels of the mitofusins, OPA1, and Drp1 cumulatively dictate the balance between mitochondrial fusion and division in different cellular contexts. For example, upregulation of Drp1 and downregulation of mitofusins results in the breakdown of mitochondria early during apoptosis (Frank et al., 2001; Landes & Martinou, 2011). At later stages of apoptosis, altered OPA1 activity causes a change in cristae structure, release of cytochrome c, and activation of caspases (Suen, Norris & Youle, 2008), indicating that inner mitochondrial membrane structure is intertwined with regulatory pathways influencing cellular life and death. Regulation of these mitochondrial‐shaping proteins occurs in different ways in various cell contexts and at many levels. This includes protein stability, protein cleavage, protein conformation via covalent modification, and protein localization via association with binding partners (Wai & Langer, 2016). Identification of these regulators may explain differences in the rates of mitochondrial fusion in distinct tissues under basal conditions. For instance, the rate of mitochondrial fusion in a neuron can be different to a muscle cell, given the specific role of mitochondrial activity in each tissue and the differences in cell polarization (Westermann, 2010).

In brief, the steady‐state levels of mitofusins are determined by their degradation by the ubiquitin–proteasome system, which in turn influences mitochondrial fusion (Cohen et al., 2008). Two independent pathways of ubiquitylation/deubiquitylation have been suggested to control activation and degradation of mitofusins (Anton et al., 2013). Two different lysine residues (K398 and K464) of the yeast Mfn ortholog, Fzo1, are specifically modified with ubiquitin by two different E3 ligases (Anton et al., 2013). An E3 ubiquitin ligase containing the F‐box protein Mdm30 (mitochondrial distribution and morphology protein 30; SCF^Mdm30^) functions in the activating pathway by attaching stabilizing ubiquitin chains (Anton et al., 2013; Escobar‐Henriques & Anton, 2013). The deubiquitylase Ubp12 selectively removes the activating ubiquitin chains and impairs outer membrane fusion which results in mitochondrial fragmentation (Anton et al., 2013). By contrast, an unknown E3 ligase, independent of SCF^Mdm30^, attaches destabilizing ubiquitin chains to Fzo1. A second deubiquitylase (Ubp2) removes the destabilizing ubiquitin chains and promotes outer membrane fusion (Anton et al., 2013). Additionally, in response to mitochondrial depolarization or cellular stress, Mfn2 is phosphorylated by Jun N‐terminal kinase (JNK) or PTEN‐induced putative kinase 1 (PINK1), which induces its ubiquitylation by the E3 ligases Huwe1 and Parkin to target Mfn2 to the proteasome (Tanaka et al., 2010; Leboucher et al., 2012; Chen & Dorn, 2013). OPA1 is uniquely regulated by post‐transcriptional and post‐translational mechanisms and exists as several different‐sized isoforms with overlapping functions. These isoforms are regulated by proteolysis and show distinct sites of cleavage (Griparic et al., 2004; Ishihara, Eura & Mihara, 2004). The mitochondrial fission mediator Drp1 is regulated by post‐translational modifications such as phosphorylation and the addition of small ubiquitin‐like modifier (SUMO) proteins (Santel & Frank, 2008), as well as by association with binding partners on the mitochondrial surface. In this review, we focus on the upstream regulation of Mfn2.

The observation that Mfn2 mRNA and protein expression was diminished in skeletal muscles of type 2 diabetics, as well as in obese patients and rats, initiated investigations into transcriptional regulators of Mfn2 (Bach et al., 2005). Mfn2 expression is upregulated under several conditions including exposure to cold, treatment with β‐adrenergic agonists (CL‐316243), and exercise. The above conditions are characterized by enhanced expression of peroxisome proliferator‐activated receptor gamma coactivator, PGC‐1α (Soriano et al., 2006), a transcriptional regulator, leading to an association between PGC‐1α and Mfn2 expression. Indeed, this association was confirmed with PGC‐1α reported to increase mitochondrial biogenesis by markedly enhancing Mfn2 mRNA and protein levels in muscle and brown adipose tissue (Puigserver et al., 1998; Soriano et al., 2006). Furthermore, PGC‐1α stimulates transcriptional activity of a 2 kb fragment of the Mfn2 promoter after transfection in several different cell types (Soriano et al., 2006). Mapping analysis of the promoter identified a region containing three putative binding sites for nuclear hormone receptors, which is critical for PGC1‐α activation. Chromatin immunoprecipitation, electrophoretic mobility shift assays and transfection analyses indicated that this specific region of the Mfn2 promoter binds to and is activated by the nuclear hormone estrogen‐related receptor ERRα, and is further coactivated by PGC‐1α (Soriano et al., 2006). However, PGC‐1α knockout mice do not show alterations in mitochondrial volume, number or size in skeletal muscles (Arany et al., 2005). Given this phenotype, it is likely that under basal conditions Mfn2 expression in PGC‐1α knockout mice remains normal. Thus, although PGC‐1α is a major regulator of Mfn2 activity under situations of high‐energy expenditure (such as cold in brown adipose tissue and muscle, fasting in liver or exercise in muscle) (Bach et al., 2005; Soriano et al., 2006), it is not thought to play a major role in the control of mitochondrial biogenesis in skeletal muscle under basal conditions.

In this regard, the role of a PGC‐1α homologue, PGC‐1β, was addressed to determine whether it affected Mfn2 expression under basal conditions. In contrast to PGC‐1α, PGC‐1β expression in several tissues is unaffected by physiological processes involving increased energy demands (Gali Ramamoorthy et al., 2015). However, PGC‐1β is expressed at higher levels than PGC‐1α in primary human skeletal muscle (Staiger et al., 2006), and muscle mitochondrial size and oxygen consumption are reduced under basal conditions in PGC‐1β knockout mice (Lelliott et al., 2006; Vianna et al., 2006). In addition, PGC‐1β expression levels are lowered in muscles of type 2 diabetics (Mootha et al., 2003; Patti et al., 2003). Similar to PGC‐1α, PGC‐1β overexpression increases Mfn2 mRNA levels in muscle cells through the activation of a 2 kb fragment of the Mfn2 promoter, mainly through ERRα coactivation (Liesa et al., 2008). PGC‐1β overexpression leads to a larger induction of Mfn2 protein levels compared to other mitochondrial dynamics proteins (Mfn1, OPA1, Drp1) and several respiratory chain subunits (Liesa et al., 2008). In addition, PGC‐1β knockout mice show reduced Mfn2 protein expression in skeletal muscle, heart, and liver suggesting that PGC‐1β is essential for maintenance of Mfn2 expression (Liesa et al., 2008). This is supported by the observation that Mfn2 expression is reduced in muscles of PGC‐1β knockout mice, whereas no changes in the expression of other essential components of mitochondrial dynamics (Mfn1, OPA1, Drp1) were detected (Liesa et al., 2008). PGC‐1β is also involved in altering mitochondrial morphology (Liesa et al., 2008). Overexpression of PGC‐1β increased mitochondrial length and mitochondrial fusion rates in wild‐type and Mfn1 knockout mouse embryonic fibroblasts (MEFs); however, in Mfn2 knockout MEFs PGC‐1β overexpression was unable to increase mitochondrial length demonstrating that Mfn2 expression was required to induce these changes in mitochondrial morphology (Liesa et al., 2008).

**Figure 3:**
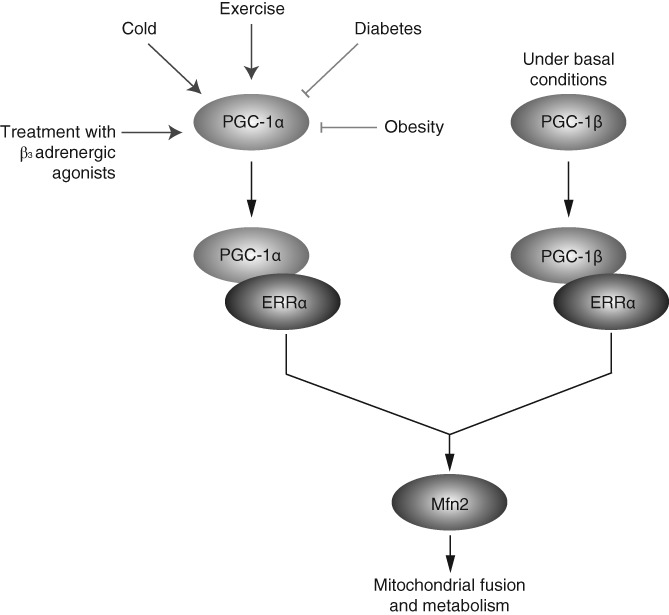
Mitofusin‐2 (Mfn2) regulatory pathway. Peroxisome proliferator‐activated receptor gamma coactivator 1α (PGC‐1α) and PGC‐1β are positive regulators of Mfn2 activity. Mfn2 is induced by PGC‐1α in response to exercise, cold exposure or β3 adrenergic agonists, whereas under basal conditions PGC‐1β activates Mfn2. This involves activation of the Mfn2 promoter through an estrogen‐related receptor α (ERRα)‐binding element.

In summary, both PGC‐1α and PGC‐1β are positive regulators of mitochondrial fusion activity under a range of physiological conditions and stimulate Mfn2 expression by targeting the Mfn2 promoter in an ERRα‐binding element‐dependent manner (Fig. 3).

## V. TISSUE‐SPECIFIC FUNCTIONS OF THE MITOFUSINS

Mitochondrial integrity including health, turnover, and repair are pivotal to human physiology. Mfn1 and Mfn2 are expressed at low levels in many tissues and display tissue‐specific expression patterns, which may explain distinct roles identified for these proteins (Sack, 2011). Mfn1 is ubiquitously expressed with higher expression levels in the heart and testis, whereas Mfn2 levels are increased in the heart, skeletal muscle, tongue, and brain (Santel & Fuller, 2001; Eura *et al.,* 2003; Santel *et al.,* 2003).

Characterizing patterns of Mfn1 and Mfn2 expression is the first step towards dissecting their tissue‐specific roles. Defects caused by mutations in Mfn2 are partly rescued by Mfn1, suggesting that a partial redundancy of function exists between the two proteins (Detmer & Chan, 2007a). Ablation of Mfn1 or Mfn2 causes embryonic lethality in mice, demonstrating their critical importance during embryonic development (Song *et al.,* 2015). Loss of Mfn2 leads to placental dysfunction as a result of defective giant placental cells, while Mfn1 knockout mice display no placental defects (Chen *et al.,* 2003). Mice with conditional Mfn2 knockout in the cerebellum show reductions in dendritic outgrowth and spine formation in Purkinje cells and reduced cell survival, whereas Mfn1 conditional knockout mice show no such defects (Chen, McCaffery & Chan, 2007). In addition, Chen *et al*. (2007) found tissue‐specific roles for mitochondrial fusion in the cerebellum and skeletal muscle. In the cerebellum, mitochondrial fusion is necessary for cell survival and for the movement of mitochondria within neurons (Chen *et al.,* 2007), whereas in skeletal muscles it is essential for protecting against the deleterious effects of mitochondrial DNA mutations (Chen *et al.,* 2010). Furthermore, loss of Mfn1 or Mfn2 results in morphologically distinct mitochondrial fragmentation (Chen *et al.,* 2003). Indeed, mitochondria harbouring only Mfn1 show higher tethering efficiency than those with only Mfn2 (Ishihara *et al.,* 2004). Consistent with this greater inter‐mitochondrial tethering capacity for Mfn1, cells lacking Mfn1 display a stronger defect in mitochondrial fusion than those lacking Mfn2 (Chen *et al.,* 2003). Taken together, these distinct but complementary functions of the mitofusins reveal the different requirements of specific tissues for mitochondrial function.

The two mitofusins are both expressed in skeletal muscle (Santel *et al.,* 2003; Zorzano, 2009). Chen *et al*. (2010) also explored the relative importance of the proteins in this tissue following postnatal conditional knockdown of each gene either alone or in combination. Knockdown of either gene did not result in a robust phenotype suggesting functional redundancy in this tissue. However, simultaneous depletion of both Mfn1 and Mfn2 resulted in a stark phenotype with grossly perturbed mitochondria and premature lethality (Chen *et al.,* 2010). Prior to the development of physiological abnormalities, the absence of both mitofusins resulted in severe depletion of mitochondrial DNA (mtDNA) levels and in the fidelity of the mitochondrial genome (Chen *et al.,* 2010). Furthermore, this loss of homeostatic mitochondrial function induced robust organ‐specific and systemic effects (Chen *et al.,* 2010). Disruption of mitochondrial fusion strongly increases mitochondrial dysfunction and lethality in a mouse model with high levels of mtDNA mutations, suggesting a protective role for Mfn2 against mtDNA mutations (Chen *et al.,* 2010).

The role of mitofusins in the heart was explored by generating conditional deletion of cardiac Mfn2 (Papanicolaou *et al.,* 2011). Cardiac myocytes lacking Mfn2 displayed a modest enlargement of mitochondria without a robust effect on basal mitochondrial respiration or cardiac function. However, the depletion of Mfn2 attenuated cardiac cell death in response to ischaemia–reperfusion injury and reduced the potential to undergo calcium‐dependent mitochondrial permeability transition (MPT) (Papanicolaou *et al.,* 2011). MPT is a tightly regulated process that is mediated by mitochondrial permeability transition pore (MPTP), a high‐conductance, calcium‐sensitive channel that induces mitochondrial depolarization and dysfunction upon opening (Haworth & Hunter, 1980; Perez & Quintanilla, 2017). MPTP is an important determinant of myocyte loss, especially during ischaemia and reperfusion injury (Di Lisa & Bernardi, 2006; Baines, 2009; Halestrap, 2009). Together, these data implicate Mfn2 as a pivotal protein for mitochondrial morphogenesis, in predisposition of cells to mitochondrial permeability transition, and in cell death (Papanicolaou *et al.,* 2011). Furthermore, this suggests that Mfn2 is important in the control of intracellular calcium stores that are involved in the cardiac response to metabolic stressors.

The function of Mfn1 and Mfn2 in endothelial cell biology has also been explored. Interestingly, both genes were induced in endothelial cells exposed to the angiogenic mitogen vascular endothelial growth factor (VEGF), and knockdown of either mitofusin decreased VEGF‐mediated migration and differentiation (Lugus *et al.,* 2011). In addition, distinct roles for the mitofusins were observed in the endothelial cells, with Mfn2 reduction exclusively blunting basal and stress‐induced levels of reactive oxygen species and Mfn1 knockdown specifically impairing VEGF signal transduction and nitric oxide production (Lugus *et al.,* 2011). As abrogation in VEGF signalling is associated with metabolic dysfunction (Schiekofer *et al.,* 2005), these data suggest a potential role of the mitofusins in vascular pathology associated with metabolic stress.

Additionally, tissue‐specific roles for Mfn2 have been identified in neuronal cells (Misko *et al.,* 2010, 2012). Loss or mutation of Mfn2 impaired mitochondrial transport in dorsal root ganglia cells, likely as a result of disrupted interaction between Mfn2 and members of the molecular complex, namely Miro and Milton, that links mitochondria to kinesin motor proteins (Misko *et al.,* 2010). Mutations in Mfn2 are causative for the peripheral neuropathy disease Charcot–Marie–Tooth type 2A (Zuchner *et al.,* 2004). Consistent with the role of mitofusins in directly regulating mitochondrial transport, patients with this disease are characterized by degeneration of only the longest peripheral axons. To date, no Mfn1 mutations have been associated with human disease.

## VI. MITOFUSIN‐2 REGULATES CELLULAR METABOLISM

Several lines of evidence support a regulatory role for Mfn2 in cell metabolism (Table 1). In this regard, the high abundance of Mfn2 observed in skeletal muscles is crucial for maintenance of myotubule structure in this tissue (Bach *et al.,* 2003). Similarly, Mfn2 repression by adenoviral antisense expression reduces glucose oxidation in cultured L6E9 rat skeletal muscle cells (Pich *et al.,* 2005). This is marked by increased glucose transport and lactate production, whereas glucose incorporation into glycogen is significantly reduced (Pich *et al.,* 2005). Additionally, the rate of palmitate oxidation is also reduced in L6E9 cells and is associated with a reduction in mitochondrial membrane potential in the presence of several oxidative substrates (Bach *et al.,* 2003; Pich *et al.,* 2005; Zorzano *et al.,* 2010). Stable knockdown of Mfn2 expression also reduces glucose oxidation and oxygen consumption in fibroblasts (Bach *et al.,* 2003). Fibroblasts carrying null mutations in Mfn2 show loss of mitochondrial membrane potential, reduced endogenous respiration, and are unable to increase respiration following ATP depletion, suggesting that cells with low Mfn2 activity rely on anaerobic glycolysis to generate energy. Thus, loss of Mfn2 function causes metabolic alterations to mitochondria characterized by reduced mitochondrial membrane potential and cellular oxygen consumption, as well as depressed substrate oxidation. In support of this notion, human fibroblasts carrying Mfn2 R364Q or A166T mutations also show metabolic alterations characterized by enhanced basal oxygen consumption, enhanced oligomycin‐insensitive respiration, and reduced mitochondrial membrane potential. However, these mutants displayed no change in the activity of the oxidative phosphorylation complexes (Loiseau *et al.,* 2007), whereas other mutations, including M21V, T105M, I213T, or V273G, did not show metabolic alterations (Loiseau *et al.,* 2007; Amiott *et al.,* 2008). The reasons for selective Mfn2 mutants showing metabolic alterations are currently unknown.

**Table 1:** Role of mitofusin‐2 (Mfn2) in cell metabolism.

Expression	Metabolic effects	References
Loss‐of‐function	Low mitochondrial membrane potential Reduction of oxygen consumption Inhibition of glucose, pyruvate, and fatty acid oxidation Low enzymatic activity of respiratory chain complexes I, II, III, and V	Bach *et al*. (2003); Chen *et al*. (2005); Pich *et al*. (2005) and Zorzano *et al*. (2010)
Gain‐of‐function	High mitochondrial membrane potential Increased glucose oxidation	Pich *et al*. (2005)
Gain‐of‐function (Mfn2 Δ602–757)	No fusion activity High mitochondrial membrane potential Increased glucose oxidation	Pich *et al*. (2005)

The mechanisms by which Mfn2 disruption alters cell metabolism is still unclear. However, studies from Mfn2 knockdown in L6E9 cells show reduced expression of several subunits involved in respiratory complexes I, II, III and V (Pich *et al.,* 2005). In addition, the enzymatic activity of respiratory complexes I+III or III is also decreased in Mfn2 knockdown cells (Pich *et al.,* 2005). This observation with knockdown of Mfn2 is in contrast to those described above for Mfn2 mutations, and is likely a consequence of reduced *versus* altered Mfn2 function or cell‐type specific differences (Pich *et al.,* 2005; Loiseau *et al.,* 2007; Amiott *et al.,* 2008). Aberrant activity of complexes I, II and III is also detected in permeabilized Mfn1 and 2 double‐knockout cells (Chen, Chomyn & Chan, 2005). Conversely, overexpression of Mfn2 in HeLa cells causes perinuclear aggregation of mitochondria, enhanced mitochondrial membrane potential, higher glucose oxidation and increased expression of several complex I, IV, and V subunits (Pich *et al.,* 2005). Thus, Mfn2 appears to play a regulatory role in mitochondrial metabolism *via* signalling pathways that result in changes in the expression of specific oxidative phosphorylation subunits.

Several lines of evidence support the suggestion that the stimulatory effects of Mfn2 on mitochondrial metabolism are independent of its fusion activity. Studies performed with a C‐terminal truncated form of Mfn2 (hMfn2 Δ602–757) that can no longer induce fusion, have shown that its overexpression in HeLa cells is marked by increased mitochondrial membrane potential and stimulation of glucose oxidation (Pich *et al.,* 2005). Overall, these data indicate that Mfn2 can activate mitochondrial metabolism and the expression of respiratory chain subunits.

## VII. MITOFUSIN‐2 AND APOPTOSIS

Mitochondria are the primary target in the early phase of intrinsic apoptosis (Martinou & Youle, 2011). Mitochondrial fragmentation during apoptosis is a result of dynamic changes in the mitochondrial localization of several proteins including Mfn2 and Drp1, and the apoptosis regulators Bcl‐2 associated X (BAX) and Bcl‐2 antagonist/killer (BAK) (Youle & Karbowski, 2005). BAX translocation from the cytosol to mitochondria is followed by extensive mitochondrial fission mediated by translocation of Drp1 to the mitochondria. Drp1 then mediates fission at the foci of BAX accumulation before caspase activation and cell death (de Brito & Scorrano, 2008). Indeed, inhibition of mitochondrial fission delays the activation of downstream caspases and apoptosis, and overexpression of either Mfn1 or Mfn2 can also delay apoptosis (Suen *et al.,* 2008).

The apoptotic proteins BAX and BAK have well‐established pro‐apoptotic roles in mitochondrial membrane permeabilization (Henry‐Mowatt *et al.,* 2004). Interestingly, these proteins can also regulate mitochondrial morphology, as BAX and BAK double‐knockout cells show fragmented mitochondria as a result of reduced mitochondrial fusion (Karbowski *et al.,* 2006). Furthermore, constitutively active Mfn2 prevents BAX translocation to the mitochondria. Our knowledge on how BAX and BAK mediate their effects on mitochondrial morphology is limited, however BAX is known to be involved in Mfn2 distribution on the outer mitochondrial membrane and BAK associates with both Mfn1 and Mfn2 (Brooks *et al.,* 2007).

## VIII. EXTRA‐MITOCHONDRIAL ROLE OF MITOFUSIN‐2

The human Mfn2 protein appears to have additional roles beyond mediating mitochondrial fusion. Mfn2 is enriched at the interface of mitochondria and the endoplasmic reticulum (ER), particularly in mitochondria‐associated membranes (MAMs), where it is involved in tethering ER and mitochondria by physically interacting with Mfn1 or Mfn2 on the outer mitochondrial membrane (de Brito & Scorrano, 2008; Naon *et al.,* 2016). In cultured cells lacking functional Mfn2, mitochondria were not tethered as tightly to the ER as normally seen in wild‐type cells (Rizzuto *et al.,* 1998). Consistent with this, Mfn2 ablation in murine fibroblasts disrupted ER–mitochondria contact sites, and led to an increased distance and change in morphology of both ER and mitochondria (de Brito & Scorrano, 2008). Furthermore, a mitochondrial ubiquitin ligase (MITOL), which mediates the addition of non‐degrading lysine 63‐linked polyubiquitin chains on mitochondrially localized Mfn2, was shown to increase the formation of ER–mitochondria contacts (Sugiura *et al.,* 2013). However, the specific mechanisms by which Mfn2 modulates ER–mitochondria contact sites are yet to be defined (Ishihara *et al.,* 2004; Koshiba *et al.,* 2004; de Brito & Scorrano, 2008). By contrast, cells with reduced Mfn2 expression have increased numbers of ER–mitochondria contact sites, high calcium transfer between the organelles, and are sensitive to apoptotic stimuli (Filadi *et al.,* 2015). In line with this, higher ER–mitochondrial tethering was associated with lower levels of Mfn1 and Mfn2 expression (Li *et al.,* 2015; Wang *et al.,* 2015). Interestingly, different roles for Mfn1 and Mfn2 in the modulation of mitochondria contacts with smooth and rough ER has been proposed (Wang *et al.,* 2015). Despite the controversies concerning the involvement of Mfn2 in the formation of ER–mitochondria contact sites, it is clear that a close apposition between the two organelles is necessary for cellular regulation.

Numerous fundamental cellular events are linked to ER–mitochondria interactions such as cell proliferation, death, autophagy, calcium signalling, biogenesis, lipid metabolism, unfolded protein response (UPR), and inflammation (Rizzuto *et al.,* 1998; Bravo *et al.,* 2012; Rowland & Voeltz, 2012; Vance, 2014). The prominent cellular pathway regulated by the close apposition between ER and mitochondria is lipid homeostasis. The role of Mfn2 in lipid synthesis was first described by Vance (1990) and Rusinol *et al*. (1994) in landmark studies, which determined that enzymes catalysing phosphatidylserine (PS), phosphatidylethanolamine (PE), and phosphatidylcholine (PC) synthesis localize to ER–mitochondria contact sites. Central to the lipid metabolism found on the MAM is the transfer of PS from the ER to mitochondria, followed by its enzymatic transformation to PE at the inner mitochondrial membrane, and transport back to the ER for conversion into PC (Vance, 2015). The ER–mitochondria contact site is also essential for formation of the autophagosome membrane, suggesting a key role of Mfn2 in the recycling of cellular contents during starvation‐induced autophagy by tethering the mitochondrial outer membrane to the ER (Hailey *et al.,* 2010). In line with this, disruption of MAM by knockdown of Mfn2 impaired the formation of autophagosomes (Hamasaki *et al.,* 2013).

In addition, Mfn2 interaction between the mitochondria and ER is crucial for the transfer of calcium released from the ER. Calcium transfer from ER to mitochondria is proposed to occur through the cytosolic chaperone glucose‐regulated‐protein 75 (GRP75), which forms a complex with the ER calcium channel inositol 1,4,5‐trisphosphate receptor (IP_3_R), and voltage‐dependent anion‐selective channel protein 1 (VDAC1) on the outer mitochondrial membrane (Rizzuto *et al.,* 1998; Szabadkai *et al.,* 2006). Calcium flux between the two organelles is necessary for regulation of respiratory chain activity, transcription of several proteins, activation of Krebs cycle enzymes, and intracellular cell signalling events in the mitochondria (Rizzuto *et al.,* 2009). Therefore, alterations in calcium flux due to lack of Mfn2 can lead to metabolic alterations signalled by calcium (Rizzuto *et al.,* 2009). Importantly, Mfn2 ablation caused defective mitochondrial calcium uptake, underscoring the importance of ER–mitochondria contact sites in calcium homeostasis, and its consequences on cellular fate (de Brito & Scorrano, 2008). Mitochondrial calcium overload also sensitizes mitochondria to apoptotic stimuli *via* prolonged opening of the permeability transition pore, which results in dissipation of the mitochondrial membrane potential, mitochondrial swelling, and the release of proapoptotic factors including cytochrome C (Crompton, 1999; Decuypere *et al.,* 2011).

Disruption of normal ER function leads to accumulation of misfolded protein in the ER lumen, which can activate the UPR (Bravo *et al.,* 2012). The UPR restores ER homeostasis by increasing synthesis of chaperones, inhibiting protein translation, and increasing degradation of misfolded proteins (Bravo *et al.,* 2012). Mfn2 has also been involved in regulating the ER stress response. Induction of ER stress in mouse embryonic fibroblasts was shown to increase Mfn2 levels (Ngoh, Papanicolaou & Walsh, 2012). Additionally, Mfn2 deficiency led to increased expression of ER chaperone proteins, resulting in amplified ER stress (Ngoh *et al.,* 2012; Sebastian *et al.,* 2012). Moreover, Mfn2 can directly interact with the ER stress‐sensing protein kinase RNA‐like ER kinase (PERK), and participates in ER stress signalling by modulating PERK‐mediated UPR signalling (Munoz *et al.,* 2013). Interestingly, silencing of PERK in mouse embryonic fibroblasts could partially rescue the fragmentation of the mitochondrial network and abnormal mitochondrial calcium content caused by loss of Mfn2 (Munoz *et al.,* 2013).

Consistent with increasing evidence that ER–mitochondria contacts are involved in regulating fundamental cellular pathways, alteration of these contacts has been reported in neurodegeneration, cancer, and obesity (Rizzuto *et al.,* 2012; Marchi, Patergnani & Pinton, 2014; Krols, Bultynck & Janssens, 2016). The Parkinson's disease‐associated proteins Parkin and PINK1 are involved in the ubiquitination of Mfn2 on damaged mitochondria (Tanaka *et al.,* 2010; Gegg & Schapira, 2011), which promotes local fission of the mitochondrial network thereby segregating damaged mitochondria for autophagic clearance (Ziviani, Tao & Whitworth, 2010). Higher levels of Mfn2 in Parkin knockout mice fibroblasts and Parkinson's disease patients were responsible for increased ER–mitochondria association (Gautier *et al.,* 2016). Similarly, drosophila PINK1/Parkin mutants displayed defective mitochondria and activated PERK‐mediated UPR signalling (Celardo *et al.,* 2016). Additionally, ER–mitochondria contact sites have been demonstrated as prime locations for Parkin‐mediated autophagy, underscoring the importance of MAM as subcellular sites for autophagy induction (Yang & Yang, 2013). Impaired function of the ER and mitochondria disrupts metabolic homeostasis, and is associated with obesity and insulin resistance (Ozcan *et al.,* 2004; Bonnard *et al.,* 2008). Moreover, selective Mfn2 ablation in pro‐opiomelanocortin (POMC) neurons in the hypothalamus resulted in loss of ER–mitochondria contact sites, ER stress‐induced leptin resistance, reduced energy expenditure, and obesity (Schneeberger *et al.,* 2013). In addition, Mfn2 expression was downregulated in mouse POMC neurons as early as 4 days after high fat diet‐induced obesity. Overexpression of Mfn2 improved the body mass, body fat level, plasma leptin, and food intake, and most importantly, attenuated markers of ER stress (Schneeberger *et al.,* 2013).

How Mfn2 enables fusion between outer mitochondrial membranes but only supports tethering (with no fusion) between mitochondrial and ER membranes remains to be elucidated. Further research should determine the relative importance and regulation of mitochondrial fusion, mitochondria–ER tethering, and mitochondrial transport in the pathophysiology of Mfn2‐associated diseases.

## IX. MITOFUSIN‐2 AND DISEASES

**Figure 4:**
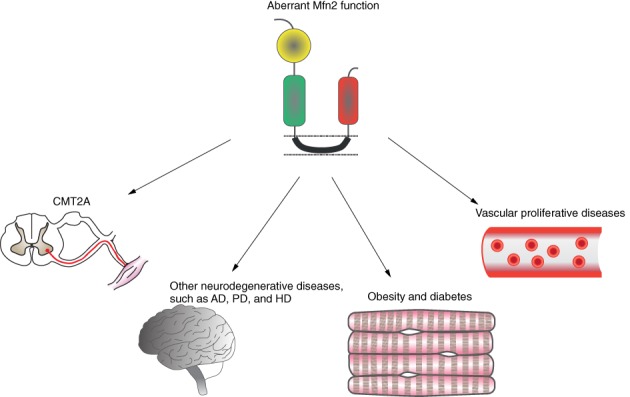
Diseases associated with mitofusin‐2 (Mfn2). Aberrant Mfn2 expression and function affects peripheral motor neurons, skeletal muscles and vascular smooth muscles leading to neurodegenerative diseases such as Charcot–Marie–Tooth type 2A (CMT2A), Alzheimer's (AD), Parkinson's (PD), and Huntington's (HD), as well as non‐neurological diseases.

It is clear that Mfn2 is a multifunctional protein whose biological functions are not just restricted to the regulation of mitochondrial shape. The number of different pathological conditions associated with Mfn2 underscores its crucial role in the regulation of cell physiology. Mutations in Mfn2 lead to defective mitochondrial dynamics, which can cause degeneration of specific neurons and result in both neurodegenerative and non‐neurological diseases (Ranieri *et al.,* 2013) (Fig. 4).

### (1) Charcot–Marie–Tooth disease type 2A

Charcot–Marie–Tooth disease (CMT) is a heterogeneous group of inherited disorders affecting the peripheral nervous system that are characterized by distal motor and sensory dysfunction (Zuchner & Vance, 2006). Worldwide, the frequency of CMT is 1 in 2500, and over the past decade, more than 40 causative genes have been identified (Barreto *et al.,* 2016). Patients with CMT vary in severity of the disease presentation ranging from moderate physical disability to severe progressive muscle weakness (Zuchner & Vance, 2006). Based on its pathology, CMT is broadly classified into two groups, demyelinating or axonal, and the most common axonal form is type 2A, accounting for up to 40% of cases (Szigeti & Lupski, 2009). The neurological spectrum in CMT2A ranges from pure motor to sensorimotor phenotypes, and affected individuals suffer tremors and muscle cramps (Pareyson & Marchesi, 2009). Strikingly, the disease is characterized by variable age of onset ranging from early childhood to adult life, suggesting that environmental factors or other genes can modulate the severity of the disease phenotypes (Muglia *et al.,* 2007). Due to the phenotypic and genetic variability, the molecular mechanisms that underlie the complex clinical manifestations of CMT have not yet been fully characterized and a lack of appropriate model systems to study the disease has hampered the identification of effective therapeutics.

Mutations in Mfn2 are primarily causal for CMT2A, but have also been reported to cause hereditary motor and sensory neuropathy VI (HMSN VI), which is an axonal neuropathy with optic atrophy (Zuchner *et al.,* 2004, 2006). To date, more than 100 different Mfn2 mutations have been reported for CMT2A (Stuppia *et al.,* 2015). The majority of disease‐causing alleles in CMT2A are missense mutations or short in‐frame deletions, and many lie in or near the GTPase domain, but mutations have been found in all domains of Mfn2 apart from the transmembrane domain (Zuchner *et al.,* 2004; Feely *et al.,* 2011). Aberrant expression of Mfn2 affects mitochondrial health and leads to axonal degeneration phenotypes in patients with CMT2A (Chapman *et al.,* 2013). Interestingly, only neurons with the longest axons are affected by degenerative phenotypes in CMT2A and defects in the axonal transport of mitochondria are proposed as a possible cause for their susceptibility (Krajewski *et al.,* 2000; Pareyson & Marchesi, 2009). In addition to the loss of peripheral nerve function, a subset of patients with CMT2A have optic atrophy, suggesting that aberrations in OPA1 and Mfn2 converge on a common pathway that can lead to overlapping clinical outcomes causing optic nerve degeneration (Zuchner *et al.,* 2004; Chung *et al.,* 2006).

Most CMT2A mutations are autosomal dominant, thus it is intriguing that a mutation of one copy of Mfn2 leads to disease. Analysis of Mfn2 mutations in mice reveals that while many of the CMT2A alleles of Mfn2 are non‐functional for fusion when expressed alone, their fusion activity can be efficiently complemented by Mfn1 (Detmer & Chan, 2007a). Thus, tissues that express Mfn1 can overcome aberrations due to loss of Mfn2 fusion activity, whereas cells with lower levels of Mfn1 expression suffer a greater relative loss of fusion activity. This may explain the selective loss of sensory and motor neurons in CMT2A where Mfn2 is normally more highly expressed.

The mechanisms by which mutations in Mfn2 can cause CMT2A are still unclear, although they could be a consequence of reduced axonal transport of mitochondria. This was first demonstrated in MEFs lacking Mfn2 (Chen *et al.,* 2003). Mitochondria in wild‐type MEFs exhibited normal retrograde and anterograde movement along the axis of the cell on radial tracks. Conversely, mitochondria in MEFs lacking Mfn2 showed spherical mitochondria with uncoordinated motion, but tubular mitochondria were still observed moving in an organized manner similar to wild‐type (Chen *et al.,* 2003). Additionally, studies in mice have shown that Mfn2 protects against degeneration of Purkinje cells in the cerebellum and dopaminergic neurons (Chen *et al.,* 2007). This may in part explain why perturbations in mitochondrial fusion lead to neurodegeneration. Although mouse models of CMT2A have been generated that directly mimic the symptoms observed in patients (Detmer & Chan, 2007a), there are still significant challenges in devising mitochondrial targeted treatments for CMT2A patients. Based on recent evidence that Mfn2 exists in both active and inactive states, pharmacologically unfolding endogenous mitofusins to promote mitochondrial tethering and fusion could prove beneficial in treating CMT2A. Indeed, Franco *et al*. (2016) designed minipeptides to manipulate the activity of Mfn2, and demonstrated that activation of Mfn2 could reverse mitochondrial defects associated with CMT2A cell models.

### (2) Additional neurodegenerative diseases

Although no specific Mfn2 mutations have been found to be causative for neurodegenerative diseases other than CMT2A and HMSN VI, emerging evidence has linked dysfunctional mitochondrial dynamics with Alzheimer's, Parkinson's, and Huntington's diseases (Wang *et al.,* 2009a *,* *b*; Su *et al.,* 2010).

Alzheimer's disease (AD) is the most common neurodegenerative disorder and is characterized by two major hallmarks: the accumulation of amyloid beta (Aβ) plaques in the cerebral cortex, and Tau‐containing neurofibrillary tangles in the brain (Ittner & Gotz, 2011). Patients present with progressive cognitive dysfunction and memory impairments, for which synaptic loss and dysfunction are considered to be major causes (Selkoe, 2002). Mitochondria play an indispensable role in synaptic development and plasticity, and abnormalities in mitochondrial dynamics lead to synaptic loss and dysfunction (Li *et al.,* 2004). Interestingly, mitochondria are redistributed away from axons in pyramidal neurons of AD patients, with alterations in the level of mitochondrial fusion and fission proteins (Wang *et al.,* 2008, 2009b). The levels of Mfn1, Mfn2, OPA1, and Drp1 are all significantly decreased (Wang *et al.,* 2008, 2009b). Impaired mitochondrial biogenesis, defective axonal transport, and increased Drp1‐mediated fission have also been observed in mouse models and in patient‐derived neurons (Calkins *et al.,* 2011; Manczak, Calkins & Reddy, 2011). In a recent study, decreased Mfn2 expression during the progression of AD was shown to be partly due to suppression by microRNA‐195 (miR‐195), and inhibition of this microRNA could prevent the decline in Mfn2 levels, thus providing a potential new therapeutic strategy for AD (Zhang *et al.,* 2016).

Parkinson's disease (PD) is the second most common neurodegenerative disease. This disease is characterized by disordered movement as a result of dopaminergic neuron loss in the substantia nigra, and non‐motor symptoms that include dementia and depression (Massano & Bhatia, 2012). Mutations in two mitochondrial genes, PINK1 and Parkin, are causal for hereditary PD, linking mitochondrial dysfunction to this disease (Kitada *et al.,* 1998; Valente *et al.,* 2004; Dodson & Guo, 2007). Mfn2 is a key ubiquitination target of Parkin (Matsuda *et al.,* 2010; Nguyen, Padman & Lazarou, 2016), and Parkin mediates the expression of PGC‐1α (Shin *et al.,* 2011), which in turn controls Mfn2 expression under stress conditions (see Section IV) (Bach *et al.,* 2003, 2005; Soriano *et al.,* 2006). Under healthy conditions, Parkin has been proposed to protect dopaminergic neurons by promoting mitochondrial homeostasis in a PGC‐1α‐dependent manner (Zheng *et al.,* 2017). Furthermore, Mfn2 has been reported to function as a receptor for Parkin in cardiac cells (Chen & Dorn, 2013; Gong *et al.,* 2015), however whether this extends beyond this cell type is unclear (Narendra *et al.,* 2008; Nguyen *et al.,* 2016). Overall, these data provide a significant link between Mfn2 regulation and PD. In addition, Mfn2 has been implicated in the function of the retromer component vacuolar protein sorting‐35 (VPS35), mutations of which are linked to familial PD (Vilarino‐Guell *et al.,* 2011; Zimprich *et al.,* 2011) and also associated with AD (Small *et al.,* 2005; Muhammad *et al.,* 2008). Targeted deletion of VPS35 in mice caused fragmented mitochondria as a result of increased mitochondrial E3 ubiquitin ligase 1 activity and consequent degradation of Mfn2 (Tang *et al.,* 2015). Importantly, stabilization of Mfn2 in the VPS35‐deletion model could prevent the loss of dopaminergic neurons (Tang *et al.,* 2015).

Huntington's disease (HD) is an autosomal dominant neurodegenerative disorder causing progressive cognitive and behavioural symptoms as a result of an expansion of CAG repeats within the huntingtin (*Htt*) gene (Reiner, Dragatsis & Dietrich, 2011). Aberrations in mitochondrial dynamics have been linked to neurodegeneration in HD (Bossy‐Wetzel, Petrilli & Knott, 2008). Overexpression of mutant HTT with 74 CAG repeats has been shown to enhance mitochondrial fission and cell death, and these phenotypes can be suppressed by overexpression of Mfn2 (Wang *et al*., 2009a). Furthermore, fragments of HTT have been shown to associate with mitochondria, interfering with their microtubule‐associated transport and thereby disrupting mitochondrial trafficking (Orr *et al.,* 2008). A possible mechanism for the imbalance of mitochondrial dynamics in HD is through aberrant transcriptional regulation, as mutant HTT binds PGC‐1α and interferes with its function (McGill & Beal, 2006), therefore possibly reducing Mfn2 expression (Fig. 3). Interestingly, *post‐mortem* brain tissue from HD patients displays significant and progressive reductions in PGC‐1α expression (Kim *et al.,* 2010). Although there is strong evidence linking perturbations in mitochondrial dynamics to neurodegenerative diseases, the precise molecular mechanisms are yet to be unraveled.

### (3) Obesity and diabetes

Changes in Mfn2 expression due to altered glucose oxidation in conditions such as diabetes, obesity, insulin resistance, exercise and weight loss, have been demonstrated both *in vitro* and *in vivo* (Bach *et al.,* 2003; Pich *et al.,* 2005). Obese and type‐2 diabetic patients display reduced Mfn2, PGC‐1α and PGC‐1β expression, whereas exercise and weight loss are linked to increased Mfn2 expression (Bach *et al.,* 2003, 2005). This is likely because acute physical exercise promotes the activation of Mfn2 transcription by PGC‐1α and ERRα (Mootha *et al.,* 2003; Patti *et al.,* 2003; Bach *et al.,* 2005). In addition, mitochondrial volume is reduced by 35% in the skeletal muscle of obese and diabetic patients, and mitochondrial morphology is disrupted in diabetic patients and obese rats. These phenotypes are likely due to reduced fusion capacity as a result of altered Mfn2 expression (Kelley *et al.,* 2002; Bach *et al.,* 2003). Furthermore, insulin administration reverses mitochondrial structural changes in diabetics (Vanhorebeek *et al.,* 2005), upregulates Mfn2 expression, and induces mitochondrial biogenesis (Pawlikowska, Gajkowska & Orzechowski, 2007). This is likely due to insulin blocking the mitogen activated protein kinase kinase 1 (MEK)‐dependent cascade as a result of Mfn2 binding to Ras and activating the phosphoinositide 3‐kinase/protein kinase B (PI3K/Akt) signalling pathway, which stimulates mitochondrial activity and in turn results in extensive mitochondrial fusion (Pawlikowska *et al.,* 2007).

### (4) Vascular proliferative diseases

Mfn2 has been shown to control the proliferation of vascular smooth muscle cells (VSMCs). Reduced Mfn2 levels are observed in spontaneously hypertensive or atherosclerosis‐prone rat VSMCs (Chen *et al.,* 2004), whereas Mfn2 overexpression decreases VSMC proliferation and sensitizes cells to H_2_O_2_‐induced apoptosis (Guo *et al.,* 2005, 2007). Moreover, Mfn2 overexpression in VSMCs causes growth arrest at the G_0/_G_1_ stage of the cell cycle, with a reduction in the number of cells in the S or G_2_/M phases (Chen *et al.,* 2004). The proposed mechanism for this growth arrest is that overexpression of Mfn2 in VSMCs physically sequesters the small GTPase Ras, thereby inhibiting the downstream Ras signalling pathway, inactivating the extracellular signal‐regulated kinase 1/2 (ERK1/2) mitogen‐activated protein kinase cascade, and eventually arresting cells in the G_0/_G_1_ phase (Chen *et al.,* 2004). In this scenario, deletion of a Ras signature motif located at amino acids 77–92 (N‐DVKGYLSKVRGISEVL‐C) eliminates the inhibitory effects of Mfn2 on ERK1/2 and subsequently on cell proliferation (Chen *et al.,* 2004). Overexpression of Mfn2 also inhibits the proliferation of VSMCs induced by exposure to oxidized low‐density lipoproteins (Guo *et al.,* 2007).

Similar observations have been made *in vivo* in response to arterial injury and in animal models of atherosclerosis. Mfn2 expression is diminished in highly proliferative VSMCs from atherosclerosis‐prone or balloon‐injured rats, and Mfn2 overexpression blocks proliferation of neointimal VSMCs after balloon injury (Chen *et al.,* 2004). This has potential clinical relevance, as proliferation of VSMCs is critical in atherosclerotic heart disease and during coronary artery restenosis secondary to balloon angioplasty. Our current knowledge points towards Mfn2 being involved in proliferative cardiovascular diseases, and suggests a possible role for Mfn2 in other proliferative disorders.

Recently, Mfn2 has become a focus in cancer research. Several studies have investigated the function of Mfn2 in different types of malignancies, including lung, gastric, liver, and urinary bladder cancers (Jin *et al.,* 2011; Wang *et al.,* 2012; Zhang *et al.,* 2013). Mfn2 can promote both pro‐apoptotic and anti‐proliferative functions. Understanding the mechanisms of mitochondrial function during tumorigenesis may provide novel insights for cancer therapeutics.

## X. OUTSTANDING QUESTIONS AND FUTURE DIRECTIONS

Despite significant advances in our understanding of how Mfn2 functions, a full understanding of its role in physiological and pathological conditions is still unclear. The development of novel experimental model systems remains key to define the mechanistic details of the multiple roles played by this molecule. Indeed, mouse and zebrafish models of CMT2A have provided crucial information on the importance of Mfn2 in normal physiology and disease (Detmer *et al.,* 2008; Vettori *et al.,* 2011; Chapman *et al.,* 2013; Bannerman *et al.,* 2016). Moreover, recent studies in small model organisms such as the fruit fly *Drosophila melanogaster* and the nematode *Caenorhabditis elegans* have been instrumental in characterizing the role of mitochondria in neuronal health (Neumann & Hilliard, 2014; Rawson *et al.,* 2014; Babic *et al.,* 2015; Neumann *et al.,* 2015; Melkov *et al.,* 2016; Morsci *et al.,* 2016; Yu *et al.,* 2016). However, despite this progress, several outstanding questions and major goals for future research remain and are outlined below.

1. Understanding the physiological role of Mfn2. Mfn2 is a multifunctional protein with roles beyond fusion. How does endogenous Mfn2 expression control mitochondrial metabolism, cell proliferation, apoptosis, and signalling? What functional Mfn2 domains are involved in maintaining these functions?
2. Identifying the modulators of Mfn2 expression. PGC‐1α and PGC‐1β are known to promote Mfn2 expression under stress and basal conditions, respectively, but it is likely that other regulators exist. Are there tissue‐specific regulators of Mfn2, are there condition‐ or disease‐specific regulators, and are these similar or different to Mfn1 regulators?
3. What intracellular signals and mechanisms regulate Mfn2 function? How is Mfn2 held in an inactive *versus* active state? Are these different confirmations tightly regulated, and are the mechanisms that control this disrupted in human disease?
4. What other molecules interact with Mfn2 and contribute to define mitochondrial distribution, shape, fusion and fission?
5. How is Mfn2 able to induce tethering with the ER, and not fusion? Understanding how this occurs will likely provide important insights into our appreciation of the mechanisms behind Mfn2 structure and regulation.
6. Only selective Mfn2 mutations are known to induce metabolic alterations. As such, defining genotype–phenotype correlations in CMT2A patients is crucial to understanding the role of Mfn2 in the disease state and to assist with the development of therapeutics.
7. Are strategies aimed at activating the fusogenic activity of Mfn2 clinically viable? In this regard, the minipeptides developed by Franco *et al*. (2016) provide a critical starting point. Characterizing the regulators of Mfn2 expression and function will provide new targets for potential therapeutics for CMT2A.
8. What is the role of Mfn2 in diseases other than CMT2A (neurodegenerative and non‐neuronal, see Fig. 4)? Does Mfn2 play a causative or consequential role in these conditions?

## XI. CONCLUSIONS

(1) Mitochondrial dynamics are essential for maintaining mitochondrial shape and transport within cells. Mitochondrial fusion and fission are mediated by several large GTPases, mainly Mfn1, Mfn2, OPA1, and Drp1, whose combined activities lead to the dynamic mitochondrial networks seen in many cell types.

(2) Association of several human genetic neurodegenerative diseases with improper regulation of mitochondrial fusion or division underscores the importance of mitochondrial dynamics in normal cell physiology (Lu, 2009).

(3) Recent studies on Mfn2 conformation and plasticity suggest that Mfn2 is tightly regulated (Franco *et al.,* 2016; Qi *et al.,* 2016).

(4) Important pieces are still missing from the puzzle that explains how mitochondrial dynamics are involved in neurodegeneration and a vast area of research still remains unexplored.

(5) Studies on Mfn2 structure, mechanism of action, and regulation are rapidly progressing, and new discoveries highlighting its regulation in different tissues will help in the identification of possible therapeutic targets for patients with related disorders.

## XII. ACKNOWLEDGEMENTS

We thank Massimo A. Hilliard and Laura Osellame for comments on this manuscript. This work was supported by NHMRC Project Grants 1099690 and 1101974 awarded to B.N., and 1106471 to M.L., and ARC Future Fellowship FT1601100063 to M.L.
